# Chrysin Inhibits Advanced Glycation End Products-Induced Kidney Fibrosis in Renal Mesangial Cells and Diabetic Kidneys

**DOI:** 10.3390/nu10070882

**Published:** 2018-07-09

**Authors:** Eun-Jung Lee, Min-Kyung Kang, Dong Yeon Kim, Yun-Ho Kim, Hyeongjoo Oh, Young-Hee Kang

**Affiliations:** Department of Food and Nutrition, Hallym University, Chuncheon 24252, Kangwon-do, Korea; reydmswjd@naver.com (E.-J.L.); mitholy@hallym.ac.kr (M.-K.K.); ehddus3290@naver.com (D.Y.K.); royalskim@hallym.ac.kr (Y.-H.K.); ohhyeongju@gmail.com (H.O.)

**Keywords:** advanced glycation end products, chrysin, glucose, glomerulosclerosis, mesangial fibrosis, TGF-β

## Abstract

Advanced glycation end products (AGEs) play a causative role in the development of diabetic nephropathy via induction of matrix protein deposition in kidneys. This study investigated inhibitory effects of chrysin, present in bee propolis and herbs, on glomerulosclerosis in db/db mice and AGEs-exposed renal mesangial cells. The in vivo study explored the demoting effects of 10 mg/kg chrysin on glomerular fibrosis in a type 2 diabetic model. Oral supplementation of chrysin inhibited the collagen fiber accumulation and α-smooth muscle actin (α-SMA) induction in periodic acid schiff-positive renal tissues of db/db mice. Moreover, treating db/db mice with chrysin diminished the level of AGEs increased in diabetic glomeruli. The in vitro study employed human mesangial cells exposed to 100 μg/mL AGE-BSA for 72 h in the presence of 1–20 μM chrysin. Glucose increased mesangial AGE production via induction of receptor for AGEs. Chrysin suppressed the induction of collagens, α-SMA, fibroblast-specific protein-1 and matrix metalloproteinases enhanced by AGE-bovine serum albumin. Furthermore, chrysin blunted transforming growth factor-β1 induction and Smad 2/3 activation in AGEs-exposed mesangial cells. These results demonstrate that chrysin attenuated accumulation of myofibroblast-like cells and matrix proteins in AGEs-laden diabetic glomeruli. Therefore, chrysin may be a potential renoprotective agent targeting glucose-mediated AGEs-associated glomerulosclerosis and fibrosis.

## 1. Introduction

Diabetic nephropathy (DN), a serious diabetic complication and a progressive kidney disease, is characteristic of glomerulosclerosis and interstitial fibrosis [1,2]. In addition, the progressive renal dysfunction causes deposition of inflammatory cells within glomeruli, which hence develops proteinuria and glomerulosclerosis [3,4,5]. Glomerulosclerosis designates scarring or hardening of the glomeruli, leading to escape of large amounts of proteins from the blood into the urine [2,6]. Kidney mesangial cells are distinctive pericytes located around the glomerular endothelium and regulate glomerular blood flow through controlling their contractile activity [7]. Hyperglycemia is the major factor responsible for structural alterations at the renal level and induces renal damage directly or through hemodynamic modifications such as increased glomerular filtration, shear stress and albuminuria [8,9]. These alterations contribute to an abnormal stimulation of resident renal cells including mesangial cells that produce more transforming growth factor (TGF)-β1 [10,11]. Hyperglycemia causes increase in the expression TGF-β genes and their receptors [12]. TGF-β1 augments deposition extracellular matrix (ECM) proteins such as several types of collagens, fibronectin and laminin at the glomerular level, thus inducing mesangial expansion and glomerular basement membrane thickening [8,13].

TGF-β is instigated by increased glucose level through inducing oxidative stress and advanced glycation end products (AGEs) production [8,14]. AGEs are glycosylated proteins or lipids non-enzymatically formed between sugars and free amino residues of proteins, lipids and nucleic acids. Protein glycosylation for AGEs in hyperglycemia is a major cause of diabetic complications [15]. Activated mesangial cells proliferate through triggering cellular mechanisms linked to production of AGEs and TGF-β [16,17] AGEs increase reactive oxygen species (ROS) generation, which subsequently induces mesangial cell hypertrophy and fibrosis [17]. The AGE toxicity may occur through interaction with the receptor for AGEs (RAGE) and their tissue deposition [18]. In addition, AGEs promote profibrotic synthesis of matrix proteins, as occurs in diabetic glomerulosclerosis and non-diabetic renal injury [14,18,19]. On the other hand, AGEs are known to modulate the expression of matrix metalloproteinases (MMPs) degrading ECM [20]. There is a correlation between the expression of MMPs and progression of renal diseases such as glomerulonephritis and tubular diseases [20]. AGEs play a causative role in the development of DN via induction of ECM accumulation [21]. Increased evidence supports that the attenuation of AGEs formation and the inhibition of RAGE activation are manifest in improving renal function [22,23]. Therefore, the AGE-RAGE interaction is a novel therapeutic target for counteracting diabetic complications [24].

**Figure 1:**
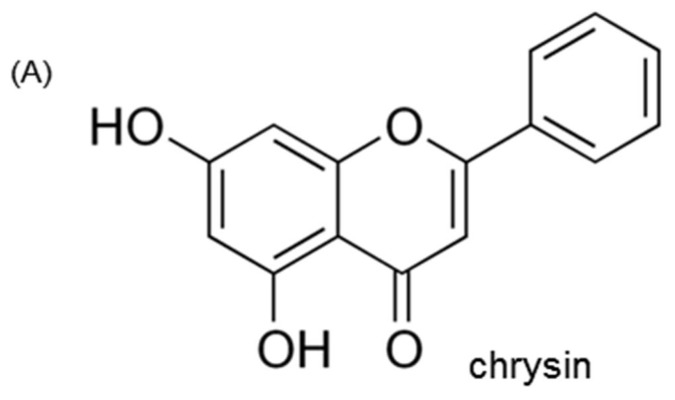

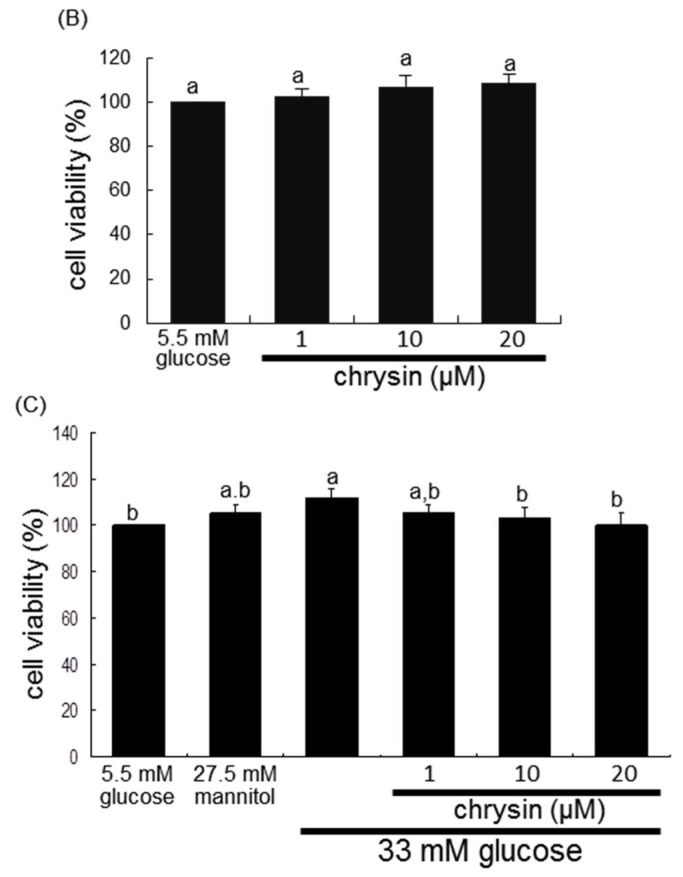
Chemical structure (**A**) and cytotoxicity (**B**) of chrysin and inhibition of glucose-exposed human renal mesangial cell (HRMC) outgrowth by chrysin (**C**). Human renal mesangial cells were challenged with 5.5 mM glucose, 5.5 mM glucose plus 27.5 mM mannitol as osmotic controls or with 33 mM glucose in the absence and presence of 1–20 μM chrysin. Values of cell viability are mean ± standard error of mean (SEM, *n* = 5) and expressed as percent cell survival relative to 5.5 mM glucose (cell viability = 100%). Values in bar graphs not sharing a same lower case indicate significant different at *p* < 0.05.

Chrysin (Figure 1A), a physiologically active flavone naturally present in passion flowers, honey and mushroom, is known to be a compound exhibiting strong pathophysiological activity such as anticancer, antioxidant, anti-inflammatory and neuroprotective properties [25,26,27]. Our previous investigation showed that chrysin inhibited diabetes-associated renal tubulointerstitial fibrosis through blocking epithelial to mesenchymal transition [28] and ameliorated podocyte injury via an inhibition of endoplasmic reticulum stress [29]. Based on the possible anti-diabetic effects of chrysin [28,29,30], this study was designed to evaluate renoprotective potential of chrysin against diabetes-associated glomerulosclerosis in glucose- or bovine serum albumin-conjugated AGE (AGE-BSA)-exposed human renal mesangial cells (HRMC) and in db/db mice. The in vitro study demonstrated that the treatment with chrysin markedly suppressed mesangial fibrosis through blocking AGE-RAGE interaction and TGF-β signaling. The in vivo study proved that oral administration of chrysin retarded glomerulosclerosis through inhibiting AGE deposition within kidney glomeruli.

## 2. Materials and Methods

### 2.1. Chemicals

Fetal bovine serum (FBS), trypsin–EDTA and penicillin–streptomycin were obtained from BioWhittaker (San Diego, CA, USA). 3-(4,5-Dimetylthiazol-yl)-diphenyl tetrazolium bromide (MTT) was purchased from DUCHEFA Biochemie (Haarlem, Netherlands). Dulbecco’s modified eagle’s media (DMEM), Nutrient Mixture F-12 Ham medium, mannitol and d-glucose, were supplied by Sigma Chemical (St. Louis, MO, USA), as were all other reagents unless specifically stated otherwise. AGE-BSA antibody was provided by Bioss Antibodies (Woburn, MA, USA). FSP-1 antibody was obtained from AbCam (Cambridge, UK). Antibodies of α-SMA, collagen 1, collagen IV, RAGE, MMP-2, MMP-9 and CTGF were provided by Santa Cruz Biotechnology (Dallas, TX, USA). MT-1 MMP antibody was purchased from R&D system (Minneapolis, MN, USA). Antibodies of TGF-β receptor I (RI), TGF-β receptor II (RII) and Smad 2/3 were obtained from Cell Signaling Technology (Beverly, CA, USA). TGF-β RI kinase inhibitor was obtained from Calbiochem (Darmstadt, Germany). Horseradish peroxidase (HRP)-conjugated goat anti-rabbit IgG, goat anti-mouse and donkey anti-goat IgG were obtained from Jackson ImmunoResearch Laboratories (West Grove, PA, USA).

Chrysin (Sigma-Aldrich Chemical) was dissolved in dimethyl sulfoxide (DMSO) for live culture with cells; a final culture concentration of DMSO was <0.5%.

### 2.2. Mesangial Cell Culture and Viability

Human renal mesangial cells (HRMC, Sciencell Research Laboratories, Carlsbad, CA, USA) were cultured at 37 °C humidified atmosphere of 5% CO_2_ in air. Routine culture of HRMC was performed in DMEM/F12 (7:1) media containing 15% FBS, 2 mM glutamine, 100 U/mL penicillin and 100 μg/mL streptomycin. HRMC in passage of 6–10 was sub-cultured at 80% confluence and used for further experiments.

To mimic diabetic glomerular fibrosis caused by chronic hyperglycemia, HRMC was incubated in 33 mM glucose-added DMEM containing 2% FBS and 8 μg g/mL insulin for 3 days in the absence and presence of chrysin. For osmotic control incubations, another set of HRMC was cultured in DMEM containing 2% FBS (+2 μg/mL insulin) and supplemented with 27.5 mM mannitol. In addition, HRMC was incubated in 0.1% BSA media containing 5–100 μg/mL AGE-BSA for 3 days. Culture media was collected and stored at −20 °C. In another set of experiments, HRMC was incubated with 5–100 μg/mL AGE-BSA up to 3 days for diabetic glomerulosclerosis.

After the 3-day incubation period under the condition of 33 mM glucose or 5–100 μg/mL AGE-BSA, the MTT assay was carried out for measuring cell proliferation. After unconverted MTT was removed, the purple formazan product was dissolved in 0.5 mL isopropanol with gentle shaking. Absorbance of formazan dye was measured at k = 570 nm with background subtraction using λ = 690 nm.

### 2.3. Western Blot Analysis

Western blot analysis was conducted using whole-cell lysates and culture media prepared from HRMC at a density of 3.0 × 10^5^ cells. Western blot analysis was also carried out using tissue extracts prepared from mouse kidney tissue extracts, as previously described elsewhere. Whole cell lysates and kidney tissue extracts were prepared by sonication in 1 M Tris–HCl (pH 6.8) lysis buffer containing 1 M β-glycerophosphate, 0.5% sodium dodecyl sulfate (SDS), 1% β-mercaptoethanol, 0.5 M NaF, 0.1 M Na_3_VO_4_ and protease inhibitor cocktail. Cell lysates and tissue extracts containing equal amounts of total proteins or equal volumes of culture medium were electrophoresed on 6–10% SDS-PAGE and transferred onto a nitrocellulose membrane. Non-specific binding was blocked by soaking the membrane in a TBS-T buffer [50 mM Tris–HCl (pH 7.5), 150 mM NaCl and 0.1% Tween 20] supplemented 3% BSA for 3 h. The membrane was incubated with an antibody of α-SMA, FSP-1, collagen I, collagen IV, AGE, RAGE, MMP-2, MMP-9, TGF-β RI and TGF-β RII. The membrane was then incubated with a secondary antibody of goat anti-rabbit IgG, goat anti-mouse IgG or donkey anti-goat IgG conjugated to horseradish peroxidase. Each protein level was determined by using Supersignal West Pico Chemiluminescence detection reagents (Pierce Biotechnology, Rockford, IL, USA) and Konica X-ray film (Konica Co., Tokyo, Japan). Incubation with β-actin antibody was conducted for the comparative control. For the measurements of the relative intensity of bands of interest, densitometric analysis of the blots was performed by Molecular Imaging Software Version 4.0.4 (Kodak, Rochester, NY, USA).

### 2.4. In Vivo Animal Experiments

Adult male db/db mice (C57BLKS/+ Leprdb Iar; Jackson Laboratory, Sacramento, CA, USA) and their age-matched non-diabetic db/m littermates (C57BLKS/J; Jackson Laboratory) were used in the present study. To measure body weight, food and drinking water intakes, mice were housed conventionally in individual stainless-steel hanging wire-mesh cages, with food and tap water provided ad libitum. Mice were kept on a 12-h light/12-h dark cycle at 23 ± 1 °C with 50 ± 10% relative humidity under specific pathogen-free conditions, fed a standard pellet laboratory chow diet (CJ Feed, Seoul, Korea) and were provided with water ad libitum at the animal facility of Hallym University. This study included db/db mice at 7 weeks of age because they begin to develop diabetes (hyperglycemia) at the age of 7–8 weeks. The animals were allowed to acclimatize for a week before beginning the experiments. Mice were divided into three subgroups (*n* = 9 for each subgroup). The first group of mice was non-diabetic db/m control mice and db/db mice were divided into two groups. One group of db/db mice was administrated 10 mg/kg BW chrysin daily via gavage for 10 weeks. Food intake, body weight and drinking water intake of db/db mice were elevated, compared to those of db/m controls [28]. On the contrary, water drinking tended to decline from 6th week after the chrysin supplementation. On the other hand, fasting blood levels of glucose and glycated hemoglobin HbA1C were measured every other week in the mouse tail veins. The chrysin challenge reduced plasma levels of glucose and HbA1C elevated in db/db mice [28]. The 24 h urine samples were collected in metabolic cages during the 10 week-chrysin supplementation. The urine volume declined in chrysin-administrated animals by ≈50% [28]. In addition, diabetic proteinuria was alleviated by oral supplementation of chrysin to the diabetic mice [29].

All experiments were approved by the Committee on Animal Experimentation of Hallym University and performed in compliance with the University’s Guidelines for the Care and Use of Laboratory Animals (hallym R1 2016-10). No mice died and no apparent signs of exhaustion were observed during the experimental period.

### 2.5. Immunohistochemical Staining

For the immunohistochemical analysis, paraffin-embedded kidney tissue sections (3 μm thick) were employed. The sections were placed on glass slides, de-paraffinated and hydrated with xylene and graded alcohol. The sections were pre-incubated in a boiling sodium citrate buffer (10 mM sodium citrate, 0.05% Tween 20, pH 6.0) for antigen retrieval. Specific primary antibody against mouse α-SMA and AGE was incubated with the tissue sections overnight. For the α-SMA localization, the sections were stained with FITC-conjugated anti-rabbit IgG. Nuclear staining was performed with 4′,6-diamidino-2-phenylindole (DAPI). For the AGE visualization, the sections were developed with 3,3′-diaminobenzidine (DAB) as a substrate to produce brownish staining and counter-stained with hematoxylin. Each slide was mounted in VectaMount mounting medium (Vector Laboratories, Burlingame, CA, USA). The stained tissue sections were taken with an optical microscope Axioimager system (Zeiss, Oberkochen, Germany) and images and their intensity were obtained for each section (Auto-measure Axio Vision program).

### 2.6. PAS Staining

The paraffin-embedded kidney specimens were sectioned at 3 μm thickness, deparaffinized, stained with PAS reagent to identify kidney structure and hematoxylin for counterstaining. The sections were placed on glass slides, deparaffinized, stained with PAS and dehydrated with absolute alcohol. The stained tissue sections were observed using an optical microscope Axioimager system and images were obtained for each section.

### 2.7. Masson’s Trichrome Staining

Mice were sacrificed by cervical dislocation under anesthesia at the termination of experimental protocols. For the histological analysis, kidney specimens were obtained at the end of the experiments. The paraffin-embedded kidney specimens were sectioned at 5 μm thick, de-paraffinized and stained with Masson’s trichrome for the light microscopic visualization of collagen fibers. The stained tissue sections were examined using an optical microscope Axioimager and images were taken for each section.

### 2.8. Immunocytochemical Staining

Immunofluorescent cytochemical staining was performed to examine the FSP-1 induction and Smad 2/3 activation of HRMC grown on 8-well chamber slides. Cells were fixed with 4% formaldehyde for 20 min and permeated with 0.1% Triton X-100 (Sigma Chemical, St. Louis, MO, USA) for 10 min on ice. Cells were blocked with 20% FBS for 1 h and a primary antibody of FSP-1 or Smad 2/3 and a secondary antibody of Cy3-conjugated IgG were applied to cells. Nuclear staining was performed with DAPI. Each slide was mounted in a mounting medium and images of each slide were taken using an optical Axiomager microscope system.

### 2.9. TGF-β1 Production

The TGF-β1 secretion from HRMC was determined by using ELISA. TGF-β1 secretion was measured in collected culture media by using a commercial ELISA kit (R&D System, Minneapolis, MN, USA).

### 2.10. Data Analysis

The results are presented as mean ± standard error of mean (SEM) for each treatment group. Statistical analyses were performed using Statistical Analysis Systems statistical software package (SAS Institute Inc., Cary, NC, USA). Significance was determined by one-way ANOVA, followed by Duncan range test for multiple comparisons. Differences were considered significant at *p* < 0.05.

## 3. Results

### 3.1. Effect of Chrysin on Glucose-Induced Mesangial Fibrosis

**Figure 2:**
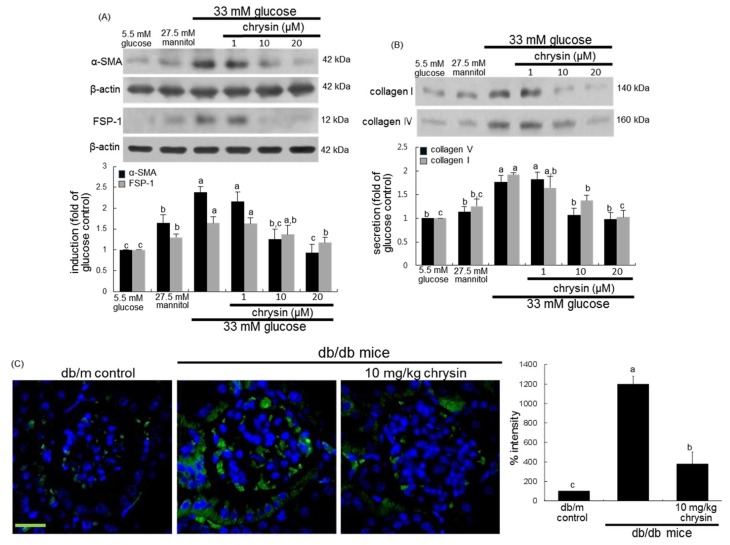
Inhibition by chrysin of glucose-induced cellular induction of α-SMA and fibroblast specific protein (FSP)-1 (**A**) and secretion of collagen I and collagen IV (**B**) in mesangial cells and α-SMA induction in mouse glomeruli (**C**). Human renal mesangial cells were challenged for 3 days with 5.5 mM glucose, 5.5 mM glucose plus 27.5 mM mannitol as osmotic controls or with 33 mM glucose in the absence and presence of 1–20 μM chrysin. Cellular induction of α-SMA and FSP-1 and secretion of collagen I and collagen IV into culture media were measured by using Western blot analysis with a primary antibody against α-SMA, FSP-1, collagen I or collagen IV (**A**,**B**). The bar graphs (mean ± SEM, *n* = 3 independent experiments) in the bottom panel represent densitometric results obtained from Image analysis. β-actin protein was used as an internal control. Diabetic mice were orally administrated with 10 mg/kg chrysin for 8 weeks. The α-SMA induction in diabetic mouse glomeruli was visualized as FITC-green staining and nuclear counter-staining was done with the blue stain DAPI (**C**). Scale bar = 25 μm. The relative staining intensity was measured and the respective values are expressed as mean ± SEM (*n* = 4 mice in each group). Values in bar graphs not sharing a same lower case indicate significant different at *p* < 0.05.

This study investigated the suppressive effects of chrysin on glucose-promoted mesangial fibrosis. There was no cytotoxicity of 1–20 μM chrysin observed (Figure 1B). In addition, the 3 day-incubation of high glucose caused the HRMC outgrowth significantly, which was blocked by treating ≥10 μM chrysin to cells (Figure 1C). As expected, glucose elevated the induction of α-smooth muscle actin (α-SMA) and fibroblast specific protein-1 (FSP-1) in mesangial cells (Figure 2A). In addition, the secretion of fibrogenic collagen I and collagen IV was enhanced in glucose-exposed mesangial cells (Figure 2B). In contrast, 1–20 μM chysin attenuated glucose-induced mesangial expression of α-SMA and FSP-1 dose-dependently (Figure 2A). The collagen production was demoted by adding chrysin to cells, in which the secretion of these collagens was restored in 20 μM chrysin-treated cells to that of glucose control (Figure 2B).

**Figure 3:**
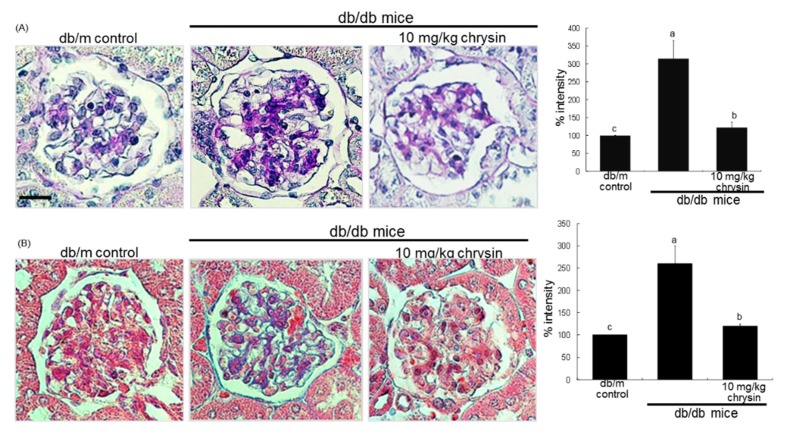
Inhibitory effects of chrysin on histological alterations (**A**) and collagen fiber deposition (**B**) in diabetic glomeruli. Diabetic mice were orally administrated with 10 mg/kg chrysin for 8 weeks. PAS staining was done in glomeruli of chrysin-treated db/db mice and nuclear counter-staining was done with hematoxylin (**A**). Masson’s trichrome staining were performed for glomerular collagen fibers (**B**) The collagen fibers were stained in blue and muscle fibers were stained in red. Each photograph is representative of at least four animals. Scale bar = 25 μm. The relative staining intensity was measured and the respective values are expressed as mean ± SEM (*n* = 4 mice in each group). Values in bar graphs not sharing a same lower case indicate significant different at *p* < 0.05.

This study further examined whether chrysin inhibited ECM accumulation and fibrosis in diabetic mouse glomeruli. The induction of green FITC-stained α-SMA was highly enhanced in db/db mouse kidneys (Figure 2C). When 10 mg/kg chrysin was orally administrated to db/db mice, the induction was markedly suppressed. Furthermore, histological evaluation revealed that diabetic kidneys exhibited increased periodic acid schiff (PAS) positivity in glomeruli (Figure 3A). In contrast, the treatment with chrysin reduced the PAS-positive staining in glomeruli of db/db mice. The intensity of PAS positivity decreased markedly in chrysin-treated mouse glomeruli (Figure 3A). On the other hand, the Masson trichrome staining showed increased the deposition of collagen fibrils in glomeruli of diabetic mice (Figure 3B). However, the administration of 10 mg/kg chrysin diminished the deposition of collagen fibrils in glomeruli with a reduction of the staining intensity (Figure 3B).

### 3.2. Effect of Chrysin on Glucose-Induced AGE Formation

**Figure 4:**
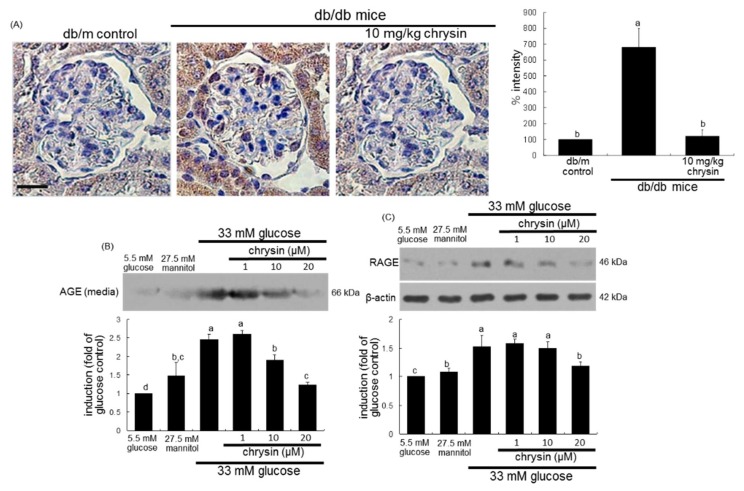
Effect of chrysin on AGE induction in diabetic glomeruli (**A**) and cellular production of AGE (**B**) and cellular induction RAGE (**C**) in glucose-exposed HRMC. Diabetic mice were orally administrated with 10 mg/kg chrysin for 8 weeks. For the AGE visualization, immunohistochemical staining for glomerular induction of AGE was conducted (**A**). Kidney tissue sections were immunostained with 3,3′-diaminobenzidine and nuclear counter-staining was done with hematoxylin. Each photograph is representative of four animals. Scale bar = 25 μm. The relative staining intensity was measured and the respective values are expressed as mean ± SEM. Human renal mesangial cells were challenged for 3 days with 5.5 mM glucose, 5.5 mM glucose plus 27.5 mM mannitol as osmotic controls or with 33 mM glucose in the absence and presence of 1–20 μM chrysin. The AGE production and RAGE induction was measured by using Western blot analyses of media or cell lysates with a primary antibody of AGE or RAGE (**B**,**C**). β-actin protein was used as an internal control. Representative blots shown are typical of three independent experiments. The bar graphs in the bottom panel represent densitometric results obtained from Image analysis. Values in bar graphs (mean ± SEM, *n* = 3 independent experiments) not sharing a same lower case indicate significant different at *p* < 0.05.

The current study examined that chrysin glucose-induced mesangial expansion and fibrosis through inhibiting AGE activation in kidneys. The AGE accumulation in the kidney glomeruli was observed in db/db mice experiencing 8 week experimental episodes, as shown by using immunohistochemical analysis. There was a heavy brown staining in db/db mouse glomerulus, indicating that AGEs were accumulated in diabetic glomerulus (Figure 4A). In contrast, the brown staining in glomeruli was diminished in chrysin-treated db/db mice. The brown staining intensity of chrysin-treated mouse glomeruli was not as strong as that of diabetic glomerulus (Figure 4A).

This study further investigated that glucose induced mesangial AGE production, which was attenuated by chrysin. The mesangial AGE production was minimal in glucose control cells, evidenced by Western blot analysis (Figure 4B). However, such production was highly enhanced in glucose-treated mesangial cells, which was consistent with the strong brown staining in db/db mouse glomeruli (Figure 4A). In contrast, 1–20 μM chrysin blunted the AGE production in a dose-dependent manner (Figure 4B). The glucose challenge induced RAGE in mesangial cells, which was attenuated by the supplementation of 20 μM chrysin (Figure 4C). Accordingly, chrysin appeared to debilitate diabetes-induced mesangial expansion by encumbering the AGE-RAGE interaction.

### 3.3. Blockade of AGEs-Induced Mesangial Expansion by Chrysin

**Figure 5:**
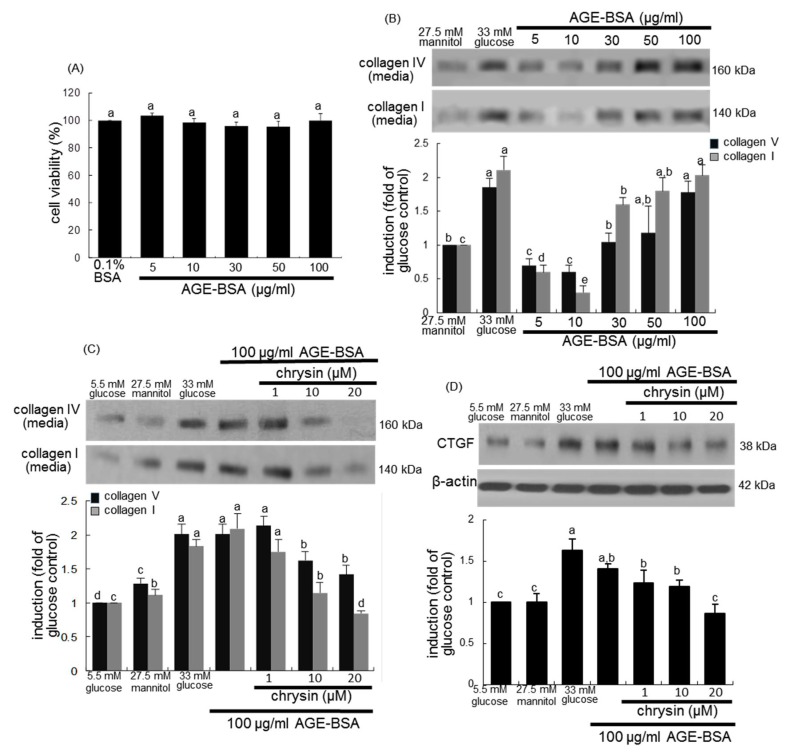
Cell viability (**A**) and collagen secretion (**B**) of 5–100 μg/mL AGE and inhibition of AGE-induced secretion of collagen I and collagen IV (**C**) and induction of CTGF (**D**) by chrysin. Human renal mesangial cells were challenged for 3 days with 5–100 μg/mL AGE-BSA in the absence and presence of 1–20 μM chrysin. Values of cell viability measured by MTT assay are mean ± SEM (*n* = 5) and expressed as percent cell survival relative to 0.1% BSA (A, cell viability = 100%). Cell extracts and media were subject to Western blot analysis with a primary antibody against collagen I, collagen IV and CTGF. β-Actin protein was used as an internal control. The bar graphs in the bottom panel represent densitometric results obtained from Image analysis. Values in bar graphs (mean ± SEM, *n* = 3 independent experiments) not sharing a same lower case indicate significant different at *p* < 0.05.

This study examined whether renal AGEs induced mesangial expansion and such induction was dampened by supplementing chrysin. The addition of ≤100 μg/mL AGE-BSA to mesangial cells did not induce cell proliferation (Figure 5A). However, as with 33 mM glucose the challenge with AGE-BSA enhanced the production of collagen I and collagen IV with increasing its doses (Figure 5B). When mesangial cells were treated with 100 μg/mL AGE-BSA for 3 days, the production of collagen I and collagen IV was promoted (Figure 5C). The addition of 20 μM chrysin to cells reduced the secretion of these collagens. It is thought that connective tissue growth factor (CTGF) cooperates with TGF-β to induce sustained fibrosis and to exacerbate ECM production in association with fibrosis-inducing conditions [31]. High glucose and AGE-BSA enhanced the induction of cellular CTGF in mesangial cells (Figure 5D). In contrast, the CTGF induction was diminished by treating chrysin to AGE-BSA-exposed mesangial cells.

**Figure 6:**
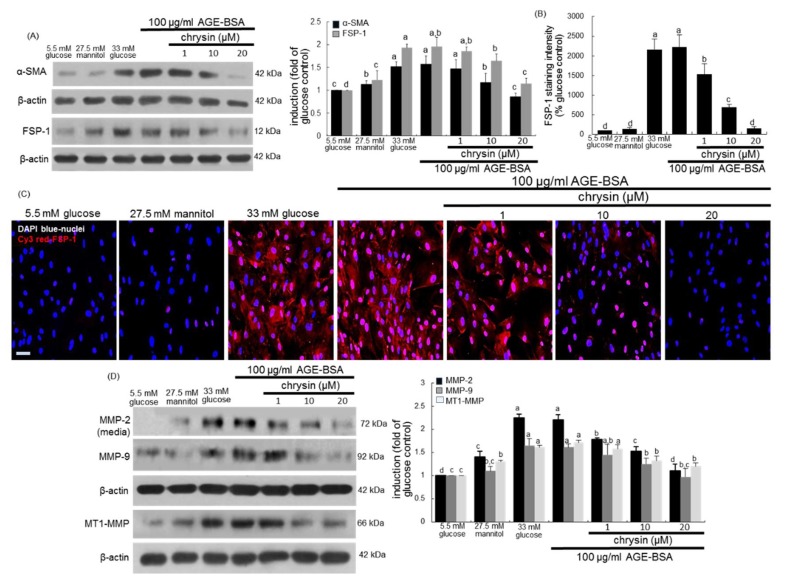
Suppressive effects of chrysin on cellular induction of α-SMA, fibroblast specific protein (FSP)-1, MM-2, MMP-9 and MT1-MMP in AGE-exposed HRMC. Renal mesangial cells were challenged for 3 days with 100 μg/mL AGE-BSA in the absence and presence of 1–20 μM chrysin. Cell extracts and media were subject to Western blot analysis with a primary antibody against α-SMA, FSP-1, MMP-2, MMP-9 or MT1-MMP (**A**,**D**). β-Actin protein was used as an internal control. The bar graphs in the right panel represent densitometric results obtained from Image analysis. The FSP-1 induction in AGE-exposed cells was immunocytochemically visualized as fluorescent Cy3 staining and nuclear counter-staining was done with the blue stain DAPI (**B**,**C**). Each photograph is representative of four different experiments and fluorescent images were taken with a fluorescence microscope. Scale bar = 100 µm. Fluorescent Cy3 staining intensity of FSP-1 was measured using an optical Axiomager microscope system (**B**). Values in bar graphs (mean ± SEM, *n* = 3 independent experiments) not sharing a same lower case indicate significant different at *p* < 0.05.

This study revealed that AGE-BSA elevated the induction of the fibroblast marker FSP-1 in mesangial cells (Figure 6A). As shown by the Cy3-immunocytochemical analysis (Figure 6B), a strong staining was observed in AGE-BSA-treated mesangial cells, indicating that AGEs may induce myofibroblast-like phenotype transdifferentiation of mesangial cells. AGEs were accumulated in diabetic glomerulus (Figure 4A). On the contrary, the cellular induction of FSP-1 was dose-dependently reduced in chrysin-treated mesangial cells (Figure 6A,B). Western blot data revealed that 100 μg/mL AGE-BSA promoted the production of a matrix-degrading MMP-2 and the cellular expression of another matrix-degrading MMP-9 (Figure 6C). The supplementation of chrysin blunted the MMP-2 secretion and the MMP-9 expression in AGE-BSA-exposed mesangial cells. In addition, AGE-BSA increased the induction of mesangial membrane type 1 (MT1)-MMP the exposure of mesangial cells, which was demoted by ≥10 μM chrysin (Figure 6C).

### 3.4. Inhibition of AGEs-Activated TGF-β/SMAD Signaling by Chrysin

**Figure 7:**
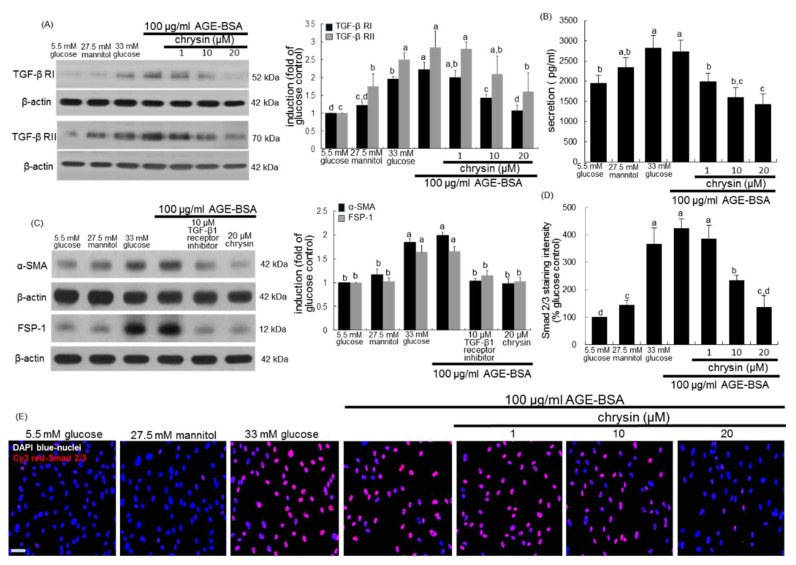
Blockade of TGF-β1 secretion, its receptor (RI and RII) induction and Smad 2/3 activation by chrysin in renal mesangial cells treated with AGE. Human renal mesangial cells were challenged for 3 days with 100 μg/mL AGE-BSA in the absence and presence of 1–20 μM chrysin or 10 μM TGF-β RI kinase inhibitor. Cell extracts and media were subject to Western blot analysis with a primary antibody against TGF-β RI, TGF-β RII, α-SMA and FSP-1 (**A**,**C**). β-Actin protein was used as an internal control. The bar graphs in the right panels represent densitometric results obtained from Image analysis. TGF-β1 in culture media was measured by using an ELISA kit (**B**). Cy3 staining was conducted for the nuclear Smad 2/3 detection with nuclear counter-staining of DAPI (**D**,**E**). Each photograph is representative of at least 4 different experiments and fluorescent images were taken with a fluorescence microscope. Scale bar = 100 µm. Fluorescent Cy3 staining intensity of Smad 2/3 was measured using an optical Axiomager microscope system (**D**). Values in bar graphs (mean ± SEM, *n* = 3 independent experiments) not sharing a same lower case indicate significant different at *p* < 0.05.

It was tested that chrysin blocked AGEs-stimulated TGF-β signaling. Western blotting showed that AGE-BSA promoted the induction of TGF-β RI and RII in mesangial cells (Figure 7A). However, the induction of these two TGF-β receptors was attenuated by ≥10 μM chrysin. When AGE-BSA was solely applied to mesangial cells, the production of TGF-β1 was enhanced as with glucose (Figure 7B). In contrast, ≥10 μM chrysin lessened the secretion of TGF-β1 elevated by AGE-BSA. The presence of 10 μM TGF-β receptor inhibitor dampened the induction of α-SMA and FSP-1 by AGE-BSA (Figure 7C).

TGF-β is known to activate its receptor-regulated Smad 2/3, allowing phosphorylated Smad 2/3/4 complex to translocate into the nucleus for fibrogenic protein expression [32]. Immunocytochemical analysis showed that AGE-BSA activated mesangial Smad 2/3 (Figure 7D,E). The nuclear translocation of Cy3-stained Smad 2/3 was observed in HRMC exposed to 100 μg/mL AGE-BSA. Nuclear Smad 2/3 activation was diminished in mesangial cells exposed to AGE-BSA in the presence of 1–20 μM chrysin (Figure 7D,E). Accordingly, chrysin dampened the TGF-β induction by AGEs and its subsequent activation of the downstream Smad family.

## 4. Discussion

**Figure 8:**
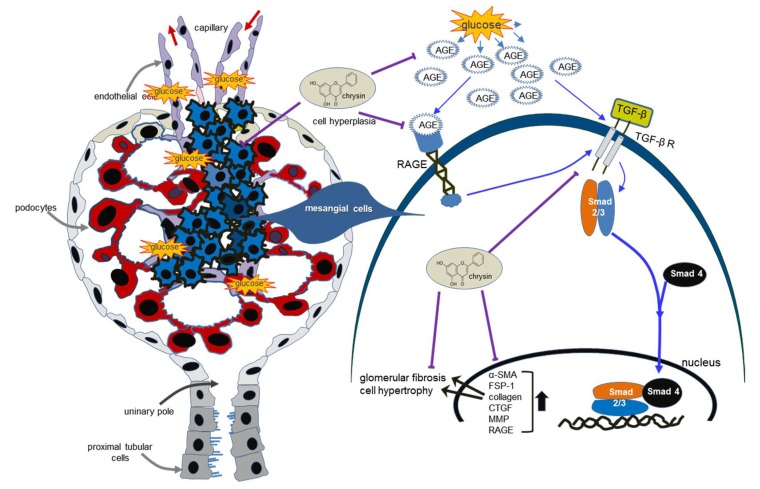
Schematic diagram showing the inhibitory effects of chrysin on kidney fibrosis in glucose/AGE-exposed renal mesangial cells and diabetic mice. Chrysin inhibited glomerulosclerosis and renal fibrosis through blocking AGEs-RAGE-TGF-β1 signaling in glucose-exposed kidneys. All the arrows indicate increase, activation or induction; ⊥ indicates inhibition or blockade.

Seven major findings were observed from this study. (1) Nontoxic chrysin at 1–20 μM attenuated glucose-induced mesangial expression of α-SMA and FSP-1 and production of collagen I and collagen IV. (2) Oral administration of 10 mg/kg chrysin encumbered the α-SMA induction and reduced the PAS-positive histological alterations and increased deposition of collagen fibrils in glomeruli of db/db mouse kidneys. (3) Chrysin dampened glucose-stimulated mesangial production of AGEs and cellular induction of RAGE. (4) The AGE deposition within glomeruli was diminished in chrysin-treated db/db mice. (5) Chrysin inhibited mesangial production and induction of collagens, FSP-1 and matrix-degrading enzymes of MMP-2, MMP-9 and MT1-MMP enhanced by 100 μg/mL AGE-BSA. (6) AGE-BSA promoted the production of TGF-β1 in mesangial cells with cellular induction of its receptors, which was diminished by chrysin. (7) The addition of chrysin to mesangial cells blunted the nuclear transactivation of Smad2/3 triggered by AGE-BSA. Collectively, this study demonstrated that chrysin markedly suppressed mesangial matrix expansion and glomerulosclerosis through inhibiting AGE deposition within glomeruli and blocking AGE-RAGE interaction (Figure 8).

Hyperglycemia is one of crucial factors responsible for the structural alterations at the renal level and induces renal damage directly or through hemodynamic modifications [9,14]. Intensive control of glucose levels is currently the mainstay of treatment for DR. Nevertheless, the development and progression of DR cannot be fully controlled. Etiological factors such as pancreatic injury disturb normal glucose metabolism. Thus, some sort of additional pharmacological therapies are required for an unmet need. Peptidyl hormones of endocrine cells origin in the gut are incretins that work to increase insulin secretion [33,34]. One investigation has shown that the incretin-based therapeutic agents with blood-glucose-lowering effects such as agonists of glucagon-like peptide 1 receptor improve pancreatic islet function, exhibit renoprotection in diabetes and also inhibit onset of the morphological abnormalities of DR [33]. This study did not investigate the manipulation of the gut-renal axis by chrysin via gavage. However, one can speculate that the therapeutic renoprotection of chrysin may be attributed, at least partly to its extrarenal and gut-originated effects. 

Structural alterations result in an aberrant stimulation of mesangial cells, leading to mesangial hyperplasia expansion [35]. This study revealed that the production of collagens that are the major component of ECM, occurred in glucose-exposed renal mesangial cells and collagen fibers were highly accumulated in PAS-positive glomeruli of db/db mice. In addition, α-SMA and FSP-1 were highly induced in glucose-induced mesangial cells and in glomeruli of db/db mice, resulting in glomerular fibrosis and glomerulosclerosis. Numerous studies have reviewed novel insights into the pathogenesis and diagnosis of DN with a special emphasis on glomerulosclerosis [2,6,36]. Activation of signaling pathways involved in diabetic glomerulosclerosis common to irreversible ECM overproduction can be manipulated for therapeutic benefit [37]. TGF-β is found in glomerulosclerosis and tubulointerstitial fibrosis caused by many renal cell types and exerts diverse biological functions via signaling pathways such as Smad and MAPK pathways [17,36,38]. CTGF is also involved in renal fibrosis by factors of high glucose and TGF-β1 and has the potential to modulate factors such as VEGF and bone morphogenic proteins in renal fibrogenesis [31,39]. The current study showed that glucose induced both TGF-β and CTGF in mesangial cells leading to collagen deposition. Thus, glomerulosclerosis and mesangial fibrosis induced by glucose may entail mesangial signaling pathway associated with the TGF-β-CTGF interaction. New therapies based on the pathophysiology of mesangial cells and targeted to TGF-β have been developed in experimental models [7,11,16].

This study found that the treatment with micromolar chrysin reduced mesangial fibrosis through inhibiting collagen deposition and α-SMA induction in glucose-stimulated mesangial cells and in PAS-positive diabetic kidneys. Several studies have shown renorotection of chrysin in experimental animal models [30,40,41,42,43]. Our previous study revealed that chrysin dampened diabetes-associated renal tubulointerstitial fibrosis through inhibiting epithelial to mesenchymal transition [28]. Chrysin ameliorates the histopathological changes such as fibrosis and inflammation in rat model of adenine-induced chronic kidney disease [43]. In this study chrysin suppressed the induction of the fibroblast marker FSP-1 in glucose-exposed mesangial cells. Accordingly, chrysin may inhibit TGF-β1-induced myofibroblast-like phenotype transdifferentiation and proliferation of mesangial cells. Since inflammatory cells such as macrophages and lymphocytes can express FGF-1, the co-localization of inflammatory infiltrates with glomerular fibrosis supports the possibility of a contribution of FGF-1 for chemotaxis and fibrosis [44]. Also, activated mesangial cells produce many inflammatory mediators leading to amplification of the injury [45]. Thus, chrysin can inhibit the infiltration of inflammatory cells in diabetic glomeruli.

AGEs are found in diabetic kidney tissues including mesangium and stimulate the production of ECM proteins, resulting in glomerulosclerosis [17,22]. This study showed that AGEs were induced in diabetic glomeruli and such induction was attenuated by oral administration of chrysin. Moreover, chrysin inhibited the increased RAGE expression and AGE secretion in glucose-exposed mesangial cells. Accordingly, the induction of profibrotic proteins of collagens, FSP-1 and α-SMA appeared to be ascribed to the increased mesangial interaction of AGE-RAGE, in which such interaction may be blunted by adding chrysin to glomerular cells. The inhibition of AGE-RAGE interaction would be a novel therapeutic target of chrysin counteracting diabetic complications [24]. Collectively, the attenuation of AGEs formation and inhibition of RAGE activation by chrysin were manifest in improving renal function including glomerular filtration through ameliorating glomerulosclerosis and mesangial fibrosis.

Nontoxic AGEs enhanced mesangial collagen production with increased expression of the ECM-degrading MT1-MMP. It should be noted that the production of MMP-2 and MMP-9 was elevated in AGEs-stimulated mesangial cells, which was dampened by supplementing micromolar chrysin. These results support the evidence that AGEs play a causative role in prompting ECM accumulation and myofibroblast migration leading to development of DN. One investigation shows that glycation of matrix proteins in mesangium results in AGE increase and reduces the matrix-degrading MMP activity of mesangial cells [46]. Unfortunately, this study did not measure mesangial matrix glycation. On the other hand, AGEs activate the angiotensin II signaling and subsequently induce TGF-β-Smad signaling in cultured rat mesangial cells [17]. Similarly, AGEs activated mesangial TGF-β and Smad 2/3 with marked induction of TGF-β receptors. The TGF-β-Smad signaling triggered by AGEs would be the therapeutic target of chrysin. Finally, AGEs induce mesangial cell injury via an ER stress-triggered signaling pathways [21].

Recent studies have introduced novel indicators that can be used for monitoring kidney function after initiation of nephroprotective treatment [47,48,49]. One study shows that serum angiopoietin-2 may be a potential predictor of acute pancreatitis severity and an indicator of renal dysfunction in the development of acute pancreatitis-renal syndrome [47]. Although this study did not monitor the serum angiopoietin-2 after chrysin treatment to diabetic mice, our previous study showed that oral administration of chrysin diminished the tissue level of angiopoietin-2 in diabetic mouse retina [30]. It was assumed that chrysin could modulate vessel outgrowth and sprouting leading to the promotion of angiogenesis and vasculogenesis. In addition, urinary neutrophil gelatinase-associated lipocalin (NGAL) is another emerging biomarker for acute kidney injury. The uNGAL level can be clinically useful in monitoring dynamic changes in kidney function for patients with acute pancreatitis [48]. It is also shown that changes in urinary NGAL during glycemic control of patients with type 2 diabetes mellitus reflect improvement of the diabetic renal dysfunction of tubules and glomeruli [49]. The measurement of urinary NGAL may be useful to monitor renal function in ongoing studies with chrysin challenge.

## 5. Conclusions

This study investigated the competence of chrysin in opposing diabetic kidney diseases characteristic of glomerulosclerosis and mesangial fibrosis. The in vitro study based on AGE injury of mesangial cells revealed that chrysin encumbered glucose-inflamed AGE-associated glomerulosclerosis and mesangial fibrosis through suppressing the induction of profibrotic proteins. The in vivo study employing diabetic kidneys showed that chrysin ameliorated diabetes-associated glomerular morphological alterations and myofibroblastic fibrosis with decreased AGE accumulation in glomeruli. AGEs-linked glomerular fibrosis may entail the interaction of AGE-RAGE and activation of TGF-Smad signaling. The capability of chrysin to deter glomerulosclerosis may be promising in hampering DN.
